# Supplementary figures and images for: High Filamin a Expression in Adrenocortical Carcinomas Is Associated with a Favourable Tumour Behaviour: A European Multicentric Study
Source: Int J Mol Sci. 2023 Nov 21;24(23):16573. doi: 10.3390/ijms242316573 (PMC10706064; doi:10.3390/ijms242316573)

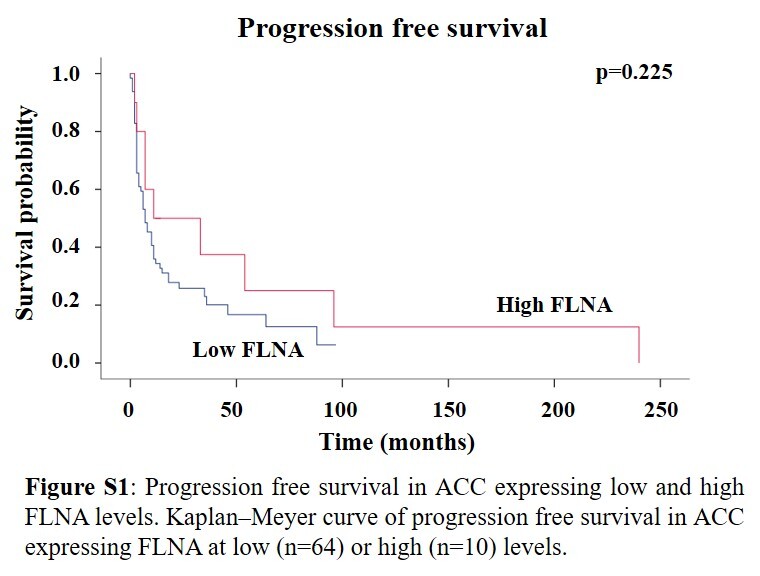

Supplement: Supplementary file 1 [file ijms-24-16573-s001.zip › figure-supplementary.jpg]
